# Slight temperature changes cause rapid transcriptomic responses in *Trypanosoma cruzi* metacyclic trypomastigotes

**DOI:** 10.1186/s13071-020-04125-y

**Published:** 2020-05-14

**Authors:** Lissa Cruz-Saavedra, Marina Muñoz, Luz Helena Patiño, Gustavo A. Vallejo, Felipe Guhl, Juan David Ramírez

**Affiliations:** 1 Grupo de Investigaciones Microbiológicas-UR (GIMUR), Departamento de Biología, Facultad de Ciencias Naturales, Universidad del Rosario, Bogotá, Colombia; 2 Laboratorio de Investigaciones en Parasitología Tropical, Facultad de Ciencias, Universidad del Tolima, Ibagué, Colombia; 3 Centro de Investigaciones en Microbiología y Parasitología Tropical (CIMPAT), Facultad de Ciencias, Universidad de Los Andes, Bogotá, Colombia

**Keywords:** *Trypanosoma cruzi*, DTUs, Temperature, Metacyclic trypomastigotes, RNAseq, Transcriptomic

## Abstract

**Background:**

Severe changes in temperature can affect the behavior and ecology of some infectious agents. *Trypanosoma cruzi* is a protozoan that causes Chagas disease. This parasite has high genetic variability and can be divided into six discrete typing units (DTUs). *Trypanosoma cruzi* also has a complex life-cycle, which includes the process of metacyclogenesis when non-infective epimastigote forms are differentiated into infective metacyclic trypomastigotes (MT). Studies in triatomines have shown that changes in temperature also affect the number and viability of MT.

**Methods:**

The objective of this study was to evaluate how temperature affects the transcriptional profiles of *T. cruzi* I and II (TcI and TcII) MT by exposing parasites to two temperatures (27 °C and 28 °C) and comparing those to normal culture conditions at 26 °C. Subsequently, RNA-seq was conducted and differentially expressed genes were quantified and associated to metabolic pathways.

**Results:**

A statistically significant difference was observed in the number of MT between the temperatures evaluated and the control, TcII DTU was not strongly affected to exposure to high temperatures compared to TcI. Similar results were found when we analyzed gene expression in this DTU, with the greatest number of differentially expressed genes being observed at 28 °C, which could indicate a dysregulation of different signaling pathways under this temperature. Chromosome analysis indicated that chromosome 1 harbored the highest number of changes for both DTUs for all thermal treatments. Finally, gene ontology (GO) analyses showed a decrease in the coding RNAs involved in the regulation of processes related to the metabolism of lipids and carbohydrates, the evasion of oxidative stress, and proteolysis and phosphorylation processes, and a decrease in RNAs coding to ribosomal proteins in TcI and TcII, along with an increase in the expression of surface metalloprotease GP63 in TcII.

**Conclusions:**

Slight temperature shifts lead to increased cell death of metacyclic trypomastigotes because of the deregulation of gene expression of different processes essential for the TcI and TcII DTUs of *T. cruzi*.
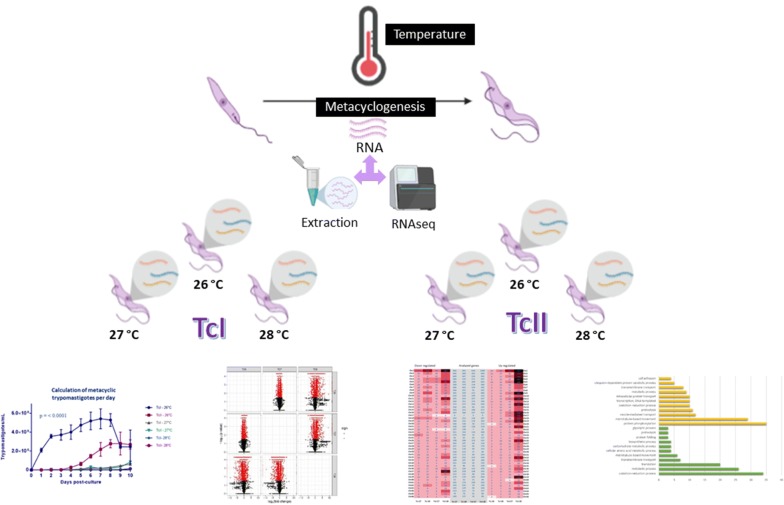

## Background

It is estimated that during the last 130 years there has been an increase of 0.85 °C in the global temperature, with the biggest changes observed during the last few decades [1]. Temperature changes affect host-pathogen interactions, vector distributions, transmission, and in some cases even the life-cycle of pathogens, mainly for vector-borne diseases such as dengue, malaria, leishmaniasis, and Chagas disease [2–11].

Triatomines (Hemiptera: Reduviidae) are the vectors of Chagas disease, an endemic pathology caused by *Trypanosoma cruzi*. Studies analyzing the effects of temperature shifts on the distribution of Chagas disease vectors have shown variable results, ranging from a possible decrease in the number of human infections to an increased risk of infection [5, 12, 13]. Studies on the impact of temperature shifts (28 °C and 30 °C) on the life-cycle of *Rhodnius prolixus*, a main vector of Chagas disease, have found that an increase in temperature decreases the transmission of the parasite, increases the ratio of feeding and reproduction, and affects the process of metacyclogenesis in the vector in the first 10 days of infection, when 26 °C is used as a control [14]. Similarly, studies of the phenol-oxidase system in the vector, which is involved in immunity against invading pathogens, have shown decreased levels at high temperatures, thus affecting both *T. cruzi* infection and vector survival [15]. This suggests that triatomine biology and the parasite life-cycle are directly affected by slight temperature shifts.

*Trypanosoma cruzi* shows high genetic variability and is classified into six discrete typing units (DTUs), associated with different epidemiological cycles, hosts, vectors, and clinical manifestations [16, 17]. In addition, this parasite has a complex life-cycle involving four stages during its passage between mammalian hosts such as humans and triatomine vectors. One of the most important steps in the life-cycle of *T. cruzi* occurs in the rectal ampulla of the vector and involves the transformation of non-infective replicative epimastigote forms into infective metacyclic trypomastigotes (MT) [18]. This process is called metacyclogenesis and involves morphological, biochemical, genetic, and transcriptional changes essential for progression of the parasite’s life-cycle [18–20]. The factors that promote metacyclogenesis are yet to be fully elucidated; however, nutritional stress increases the levels of adenylate cyclase expression and cAMP concentrations stimulate the expression of genes involved in autophagy, which is essential for the progression of this process. Some metabolic changes that have been detected in metacyclogenesis are related to the presence of oxidized proteins, the activation of enzymes involved in the metabolism of carbon and nitrogen as a source of energy, and the activation of mitochondrial enzymes, such as cytochrome, in response to nutritional stress [21–23]. Some of the changes in the parasite during this process are related to structural modifications of the kinetoplast, elongation of the nucleus, and an increase in heterochromatin, together these changes relate to the decrease in mRNA expression in MT forms [18, 24]. The success of the metacyclogenesis process depends on the expression of certain specific genes, such as those encoding methionine peptidase I (Met I), surface metalloprotease GP82, surface metalloprotease GP90, and MT-specific proteins, as well as other genes such as the *Tclmp4* gene associated with a ribonucleoprotein involved in the processing of the S40 subunit, which is important in progression of the cell-cycle [25, 26]. Transcriptomic analysis using RNAseq technology has been useful in understanding the gene remodeling that occurs in *T. cruzi* during infection, in identifying genes expressed differentially between the three stages of this parasite, and in evaluating the gene profiles between virulent and non-virulent clones [27–31]. However, until now, the transcriptional profiles of *T. cruzi* have not been evaluated when MT are exposed to different temperatures. Therefore, the objective of this study was to assess whether slight and short-term changes in temperature affect the gene transcription of *T. cruzi* MT.

## Methods

### Epimastigote culture

Cultures of epimastigotes of the strains MHOM/CO/04/MG (TcI) and MHOM/BR/53/Y (TcII) were maintained by weekly passage in liver infusion tryptose (LIT) medium supplemented with 10% fetal bovine serum. These strains were selected as these have been previously used in *in vivo* studies in murine models and triatomines, they are fully characterized and provide a suitable set of biological strains for *in vitro* studies [14]. For verification of the DTU, DNA extraction was performed from cultures of epimastigotes in logarithmic phase for both strains using the DNeasy kit (Cat. # 69504; Qiagen, Hilden, Germany), followed by conventional PCR directed to the spliced-leader intergenic region (SL-IR) as reported elsewhere [32]. The obtained products were subjected to electrophoresis on a 2% agarose gel, with expected band sizes of 300 bp for TcII and 350 bp for TcI.

### Calculation of MTs per day

To determine the day at which the number of MTs emerged, we calculated the number of MTs as previously described [33]. Epimastigotes in exponential growth phase, genotyped previously as TcI (MHOM/CO/04/MG) and TcII (MHOM/BR/53/Y), were washed twice with phosphate-buffered saline (1× PBS) and centrifuged at 10,000× *rpm*, then 1 × 10^7^ epimastigotes were cultivated in LIT medium supplemented with 5% fetal bovine serum at temperatures of 26 °C, 27 °C and 28 °C. These temperatures were selected based on an estimated temperature increase of 0.85 °C during recent decades and a previous report in *R. prolixus* [14]. To avoid a bias related to a possible inadequate initial concentration of epimastigotes, all tests were carried out from the same initial inoculum and placed in different incubators according to the required temperature. The cultures were kept for 10 days, considering that we wanted to obtain pure MT cultures. The verification of temperature maintenance in the incubators was carried out twice a day during the entire study period. The concentration of parasites was calculated using a Neubauer chamber, in order to determine the epimastigotes and trypomastigotes that were viable; the mobility of parasites was used as a discrimination parameter and only mobile forms were included in the count. The number of epimastigotes (EP) and metacyclic trypomastigotes (MT) was determined microscopically on slides fixed with 100% methanol with 10% Giemsa stain. For each slide, 300 fields were analyzed under an optical microscope (40×). Three biological replicates for each experiment were included, as well as three technical replicates for each biological replicate to decrease any operator errors.

### Statistical analysis

The data relating to the concentration of parasites and the determination of stages were tabulated in Microsoft Excel. The determination of stages was normalized as a percentage that included two factors: the percentage of MT and the percentage of EP. These data were applied to the concentration of parasites to determine the specific concentration for each morphological stage in each individual experiment. To evaluate whether the data showed a normal distribution, a Shapiro-Wilk test was carried out, followed by (provided the obtained data did not follow a normal distribution) a Kruskall-Wallis test and the analysis of Dunn’s multiple comparisons to determine the day of emergence of metacyclic forms (EMD). This day was selected as the first day when statistically significant changes were observed in the concentration of MT compared with the control (day 0). The data were also used to generate a calculation of MTs per day. Finally, the comparison between DTUs was performed using a non-parametric Friedman test followed by an analysis of multiple comparisons. All analyses were performed using GraphPad Prism 7.4 software using *P* < 0.05 as the cut-off for significance.

### Purification of metacyclic trypomastigotes

To evaluate the expression of MT genes during EMD when the parasites were subjected to different temperatures (26 °C, 27 °C and 28 °C), RNA extraction from TcI (MHOM/CO/04/MG) and TcII (MHOM/BR/53/Y) was performed. As we previously reported [33], cultures of *T. cruzi* contain a mix of EP and MT stages during EMD. To obtain a pure MT sample, we developed sepharose ion exchange chromatography according to our previously reported protocol [33]. After which, the samples were washed twice with 1× PBS and the stage was verified under a microscope.

### RNA extraction and sequencing

In order to stabilize the MT after purification by means of sepharose-DEAE resin chromatography, the MT obtained were washed twice with 1× PBS, and incubated in LIT medium without supplements for 2 h. MT purified by sepharose ion exchange chromatography were subjected to RNA extraction. The RNA was extracted from 24 samples (i.e. two DTUs at three temperatures, with two biological replicates and two technical replicates) using a RNeasy Plus Mini Kit (Qiagen) following the manufacturer’s protocol. The quality of the RNA obtained was evaluated on an agarose gel and the concentration, as well as other parameters such as the 260/280 index and the 230/260 index, were measured by nanodrop spectrophotometry. The RNAs that showed typical RNA bands, had concentrations higher that 1 mg/ml, and a 260/280 index close to 2 were selected for total RNA sequencing.

The selected RNAs were sent to Novogene Bioinformatics Technology Co., Ltd. (Beijing, China) for sequencing using the Illumina HiSeq X-TEN platform. The strand-specific TrueSeq RNAseq Library Prep with an insert size of 350 bp was selected to prepare the RNA libraries, and the size of each read was 2 × 150 bp. The read quality was verified using fastqc software (http://www.bioinformatics.babraham.ac.uk/projects/fastqc/). In summary, parameters such as per base quality score, per base sequence GC content, Kmer content (among others) were evaluated.

### Mapping and transcript quantification

The raw sequence reads, for the 24 transcriptomes included in this study, had an average of 70,630,112 bases and a standard deviation of 20,976,808.33 bases. The results for each of the treatments and replicates are available in Additional file 1: Table S1.

The fasta file for the *T. cruzi* Sylvio X10-1 genome was downloaded from the Eupath TriTryp database (https://tritrypdb.org/tritrypdb/) and Bowtie version 2 software was used as a reference index (Additional file 2: Table S2). Then 24 paired-end samples were individually aligned using TopHat version v2.1.0 and default parameters [34]. Similarly, the gtf file deform annotated genome of *T. cruzi* Sylvio X10-1 was downloaded from the Eupath TriTryp database and was used to perform transcript assembly from the reads obtained from the alignment by TopHat (Additional file 2: Table S2). The software Cufflinks version v2.0.2 (http://cole-trapnell-lab.github.io/cufflinks/) was used for this task and the “u” parameter (multi-read correlation) was included. Once the mapping process was completed, the union of the gff files obtained using the Cuffmerge tool of Cufflinks was made using the “g” and “s” options, and different files were created to analyze the differences in expression between replicates, different temperatures for the same DTU, and finally, between DTUs at the same temperature [34].

### Differential expression and ontology analysis

Cuffdiff was used to evaluate the differential expression of genes between DTUs and temperatures [34]. The normalized expression was calculated using FPKM (fragments per kilobase of exon per million fragments mapped), and comparisons between genes expressed at 27 °C and 28 °C and the 26 °C control were made for each of the DTUs. Genes that presented a Q-value (*P*-value corrected) less than or equal to 0.05 were considered to be differentially expressed. The same protocol was followed to evaluate the differential expression between replicates of samples and DTUs for each of the temperatures. The *CummeRbund* software package in R was used to visualize the output files obtained from the Cuffdiff analysis and for the generation of graphs [35]. To make a biological inference and determine the protein products encoded for each of the DEGs, and to analyze the enzymes that could be encoded by these same genes, the list of upregulated and downregulated genes obtained by the statistical analysis of Cuffdiff were analyzed using the EupathDB TriTryp online tool (https://tritrypdb.org/tritrypdb/), and the lists of DEGs for each treatment were submitted. The output files were selected based on the analysis of the gene ontological terms (GO terms) for each of these genes, and the tables obtained were recorded in Microsoft Excel for further analysis [36, 37]. Dynamic tables were constructed to quantify the number of upregulated and downregulated genes for each of the GO terms and the results were used to construct graphs. Ten biological process GO terms with the greatest number of genes were selected.

## Results

### Increase in temperature affects the emergence of metacyclic trypomastigotes in *Trypanosoma cruzi* I and II *in vitro*

**Fig. 1:**
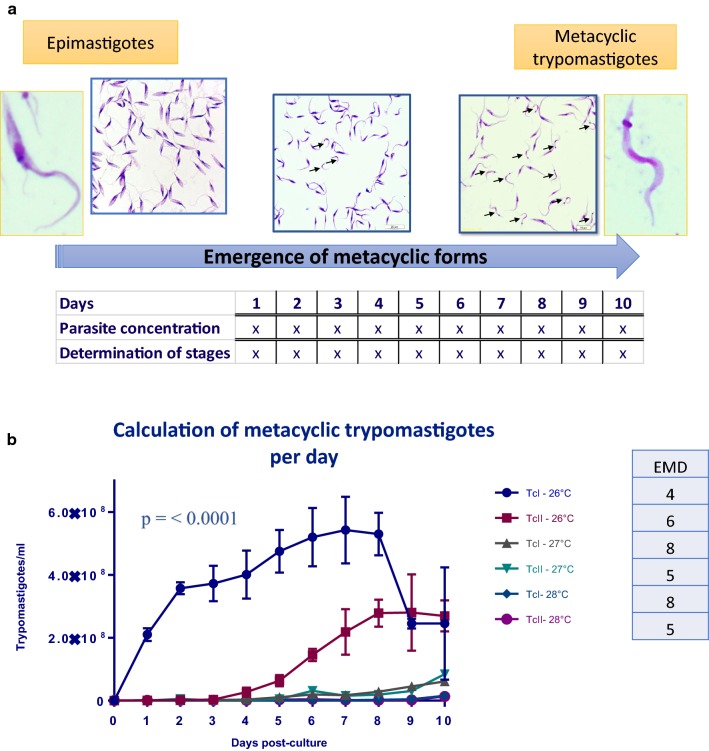
Increase in temperature affects the emergence of metacyclic trypomastigotes in *Trypanosoma cruzi* I and II *in vitro.***a** Process of generation of emergence of metacyclic curves for *T. cruzi*. **b** Calculation of metacyclic trypomastigotes per day of all discrete typing units (DTUs) and temperatures. The observed *P*-value corresponds to the comparison between DTUs and temperatures. The emergence of metacyclic forms day (EMD) for each of the DTUs is shown next to the legends. The arrows indicate the nuclei of MTs

**Fig. 2:**
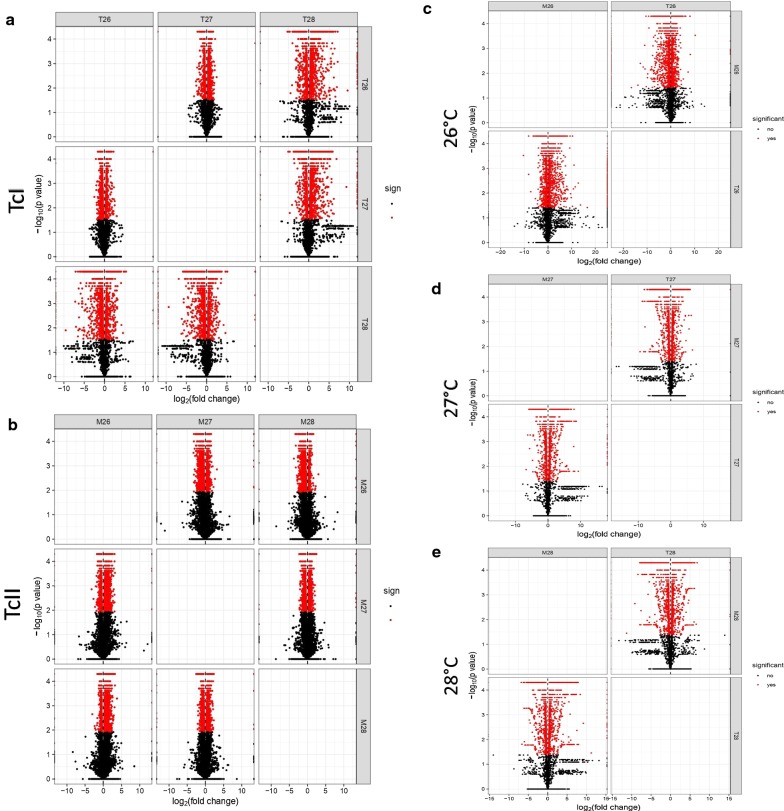
Volcano matrix of DEGs in the metacyclic trypomastigotes of *Trypanosoma cruzi* I and II. The differentially expressed genes (DEGs) are shown in red. Fold changes are shown on the X-axis and *P*-values are shown on the Y-axis. **a** DEGs for TcI at different temperatures. **b** DEGs for TcII at different temperatures. **c** DEGs for TcI and TcII exposed to 26 °C. **d** DEGs for TcI and TcII exposed to 27 °C. **e** DEGs for TcI and TcII exposed to 28 °C

The emergence of MT was evaluated in *T. cruzi* MHOM/CO/04/MG (TcI) and MHOM/BR/53/Y (TcII) cultures over 10 days (Fig. 1a). Nutritional stress is one of the main factors that promotes metacyclogenesis in *T. cruzi*, and previous studies have demonstrated the presence of MT in cultures grown in LIT medium when a high concentration of parasites was used as the inoculum. We used these previously described culture conditions and protocol to calculate the MT per day in this study [33]. No MT were observed on day 0, but low concentrations of MT were detected from day 1 in most of the treatment conditions, except for TcI incubated at 27 °C (MT observed from day 2). The highest concentrations of MT (6 × 10^8^ trypomastigotes/ml on average) were observed on day 7 for TcI incubated at 26 °C, whereas the concentration of MT for TcII under the same conditions was 2.8 × 10^8^ trypomastigotes/ml on day 8 (Fig.1b, c). The concentration of MT decreased at temperatures of 27 °C and 28 °C for both DTUs, with a maximum of 6.0 × 10^7^ and 1.7 × 10^7^ trypomastigotes/ml being detected, respectively, with the highest concentrations of MT observed on day 10 (Fig. 1a, b, e). Similar behavior was observed for TcII incubated at 27 °C and 28 °C, with maximum concentrations of 8.5 × 10^7^ and 1.3 × 10^7^ trypomastigotes/ml on day 10 (Fig. 1b, d, e). As previously mentioned, MT concentrations were higher for TcI compared with TcII in most of the experiments (Fig. 2c, d, e); however, we found the opposite behavior for TcII at 27 °C on day 10, with higher concentrations of MT compared with TcI at 27 °C; despite this, no statistically significant difference was observed on this day. We performed normality analysis using the Shapiro-Wilk test for the data obtained from each of the treatments and the results showed that some samples did not follow a normal distribution. We therefore used non-parametric analysis for statistical evaluation of our data. To determine the day of emergence of metacyclic forms (EMD), we performed a Kruskall-Wallis test followed by an analysis of Dunn’s multiple comparisons, using day 0 (0 trypomastigotes/ml) as the control. The EMD for the TcI cultured at 26 °C occurred 4 days post-culture (DPC), compared with 6 DPC for TcII at the same temperature (Fig. 1b). When the temperature was increased to 27 °C, the EMD occurred at 8 DPC for TcI and 6 DPC for TcII, and at 28 °C, the EMD was 8 and 5 DPC for TcI and TcII, respectively. Samples from the EMD were used for RNA extraction for each of the treatments (Fig. 1c, d, e). Differences between the temperatures and DTUs were analyzed by Friedman’s non-parametric tests and the results revealed statistically significant differences between the treatments (*P* < 0.0001, *df* = 6). To analyze these differences in greater detail, Dunn’s multiple comparisons were performed and no differences were detected between the DTUs treated at the same temperature. However, following Kolmogorov-Smirnov analysis, a difference between the TcI at 26 °C and TcII at 26 °C treatments was detected (*P* = 0.0233). Furthermore, a difference was detected between the parasites treated at 26 °C (control) and 28 °C for both DTUs (*P* ≤ 0.001).

### Changes in temperature increase and decrease gene transcription in MT of *T. cruzi* I and II

The concentration, purity and integrity of the extracted RNAs was verified, only the RNAs that passed the quality threshold for all these parameters were sent to sequencing. Additional file 3: Figure S1 shows the integrity RNA bands in agarose gels, RNA concentrations and the 260/280 index. The RNAs from two biological replicates and two technical replicates for each treatment were assembled and compared with the reference genome Sylvio/X10 (Additional file 2: Table S2). No differences were detected between the replicates of TcI and TcII at any of the temperatures (Additional file 4: Figure S2a, b). Differential expression analysis did not show strong variability between the genes expressed among replicates for many of the genes according to the Q-values (*P*-value corrected) *P* ≥ 0.05 when Cuffdiff analysis was performed.

A total of 11,154 genes were compared for TcI at 26 °C and 27 °C, and 11,185 genes were compared for TcI at 26 °C and 28 °C. For TcII the comparison between 26 °C and 27 °C included 5884 and in the case of 26 °C and 28 °C the number of genes corresponded to 13,187. On the other hand, the comparison between DTUs at 26 °C had 10,984 genes, for 27 °C there were 9300 genes, and finally, for 28 °C 9777 genes. Differential expression analysis revealed that the MT of TcI and TcII exposed to different temperatures showed differences in the expression of genes when compared with the control at 26 °C by Cuffdiff analysis (Fig. 2a, b, Additional file 5: Table S3). Similarly, differences in gene expression were assessed for DTUs at each of the temperatures (Fig. 2c–e); however, a smaller number of genes were observed when differences between MT treated with different temperatures but belonging to the same DTU were analyzed. This characteristic was even more marked when the comparison was made between DTUs that were exposed to 27 °C and 28 °C (Fig. 2d, e).

**Fig. 3:**
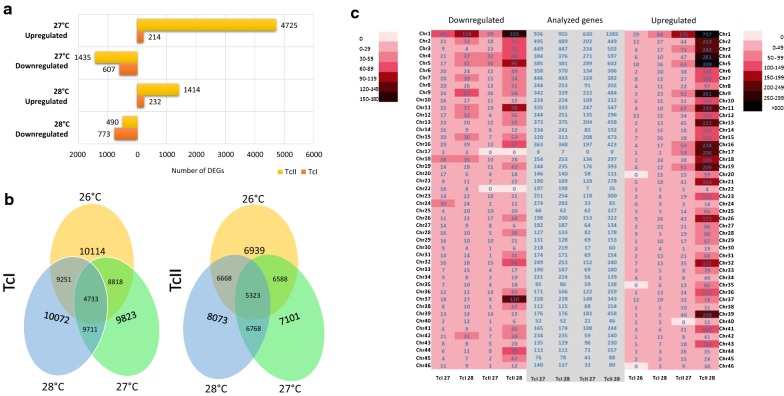
DEGs in the metacyclic trypomastigotes of *Trypanosoma cruzi* I and II. **a** Number of differentially expressed genes (DEGs). The graph shows the number of downregulated and upregulated genes for each of the exposure temperatures and indicates the total number of genes under these conditions (next to bars) and the number of genes per fold-change. **b** Venn diagram with DEGs shared between temperatures treatments for each DTU. **c** Heat map showing downregulated and upregulated genes per chromosome and the number of genes analyzed in each comparison

The TcI DTU had a greater number of downregulated genes in MT exposed to temperatures of 27 °C and 28 °C compared with the control at 26 °C, different to what was observed for 27 °C compared with 28 °C. The number of genes upregulated in TcII MT was higher for parasites exposed to 28 °C than 27 °C compared with the TcII control at 26 °C (Fig. 3a). For TcII DTU, the response to increased temperature was mainly marked by an upregulated gene expression, where the highest number of upregulated genes was observed for TcII at 28 °C (Fig. 3a).

We evaluated the presence of shared genes between different temperature treatments for each DTU. Out of a total of 10,114 expressed genes, TcI at 26 °C shared 8818 genes with TcI at 27 °C and 9251 genes with TcI at 28 °C, and TcI at 27 °C and TcI at 28 °C shared 9711 genes (Fig. 3b). The number of genes shared between TcII at 26 °C and 27 °C was 6588, and 6668 genes were shared with TcII at 28 °C, the number of genes shared between TcII at 27 °C and 28 °C was 6768. The temperature at which the greatest number of genes were expressed for TcI DTU was 28 °C (Fig. 3b).

We evaluated whether there were differences between the number of upregulated and downregulated genes among the different chromosomes, and quantified the differentially expressed genes (DEGs) in each of the chromosomes for each DTU when exposed to a range of temperatures. Chromosome 1 had the highest number of upregulated and downregulated genes for the two DTUs at all of the temperatures evaluated. Many of the DEGs were found in the MT exposed to 28 °C, with DTU TcII presenting the greatest number of changes and showing an interesting pattern of downregulation, with 165 genes downregulated on chromosome 1, 96 genes downregulated on chromosome 5, 120 genes downregulated on chromosome 37, and 98 genes downregulated on chromosome 11, in addition to various changes in the remaining chromosomes. Similarly, when the upregulated genes were evaluated by chromosome, TcII at 28 °C had an average of 100 to > 300 genes expressed in the majority of chromosomes from 1 to 21; however, the greatest number of upregulated genes corresponded to TcII at 27 °C, and no upregulated genes were observed for TcI at 28 °C, as previously observed (Fig. 3c).

### Differentially expressed genes were associated with molecular processes that play an important role during thermal stress

**Fig. 4:**
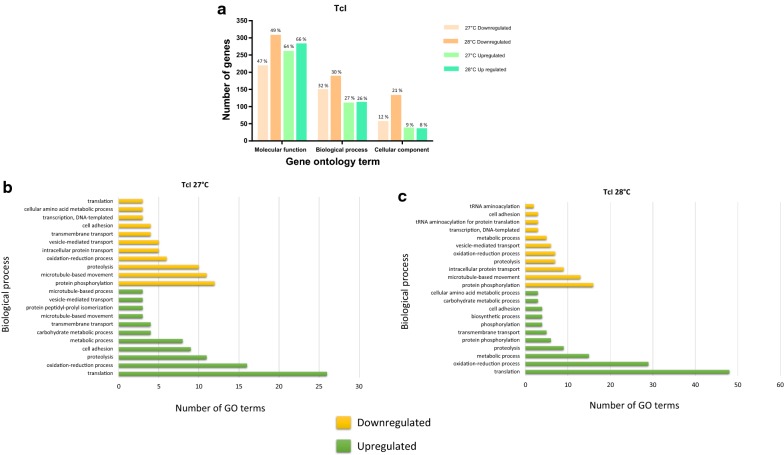
Gene ontology terms associated with the DEGs for *Trypanosoma cruzi* I. **a** Number and percentage of downregulated and upregulated genes for each discrete typing unit (DTU) and the gene ontology (GO) terms. **b** Ten GO terms with the greatest number of differentially expressed genes (DEGs) for biological processes in the MT of TcI exposed to 27 °C. **c** Ten GO terms with the highest number of DEGs for biological processes in the MT of TcI exposed to 28 °C

The IDs for each of the downregulated and upregulated genes in each of the treatments evaluated here were submitted to the Tritryp gene database of EupathDB and gene ontology analysis was performed for each DTU and temperature (Additional files 6, 7, 8: Tables S4, S5, S6). Of the downregulated genes, 607 for TcI at 27 °C and 773 for TcI at 28 °C were subjected to analysis; of these, a total of 463 and 625 GO terms, respectively, were obtained. Large percentages of the genes were associated with molecular processes. Of note, many genes related to oxidoreductase activity in TcI at 28 °C were downregulated, which is an important part of the metacyclogenesis process, and as a consequence, of the emergence of MTs. Similarly, when the terms associated with biological processes were evaluated, downregulation of genes involved in oxidation-reduction processes was observed at both temperatures (Fig. 4b, c). A total of 214 and 232 overexpressed genes in TcI at 27 °C and 28 °C, respectively, were evaluated for the ontological terms. The results for this group of genes showed a total of 406 GO terms for 27 °C and 427 for 28 °C. The highest percentage of ontology terms corresponded to molecular processes, and these results were similar to those found for downregulated genes in TcI (Fig. 4a). Ontology terms relating to proteolysis processes and the phosphorylation of proteins were also observed at both temperatures, as well as genes relating to the motor activity of microtubules (Fig. 4b, c).

**Fig. 5:**
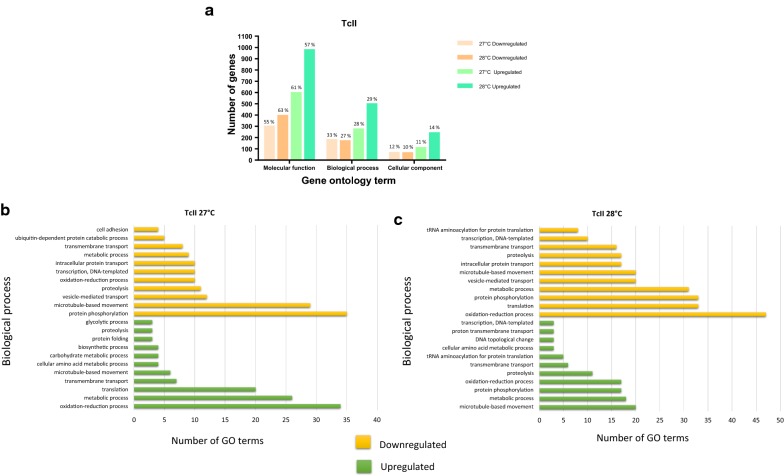
Gene ontology terms associated with the DEGs for *Trypanosoma cruzi* II. **a** Number and percentage of downregulated and upregulated genes for each discrete typing unit (DTU) and the gene ontology (GO) terms. **b** Ten GO terms with the greatest number of differentially expressed genes (DEGs) for biological processes in the MT of TcII exposed to 27 °C. **c** Ten GO terms with the highest number of DEGs for biological processes in the MT of TcII exposed to 28 °C

The most differentially upregulated and downregulated genes were associated with molecular processes based on the GO ontology for both temperatures used in TcII. A total of 1435 and 490 downregulated genes were obtained at 27 °C and 28 °C, respectively; these were associated with 541 and 625 GO terms, respectively. Downregulated genes associated with GO terms involved with oxidation-reduction processes and oxidoreductase activity were found at both temperatures (Fig. 5b, c). A total of 1414 and 4725 genes were upregulated in TcII at 27 °C and 28 °C, respectively; of these, we found associations with 977 and 1713 GO terms, respectively, including terms associated with the upregulation of transport mediators by vesicles (Fig. 5b, c). Some of the processes with the largest number of DEGs are described below.

### Metabolism

A high degree of regulation was observed for genes involved in the metabolism of lipids in the MT when exposed to different temperatures. The treatment with the most DEGs are involved in lipid metabolism was TcII at 27 °C with 10 downregulated genes, followed by TcI at 28 °C with 8 downregulated genes and TcI at 27 °C with 6 downregulated genes when compared to TcI at 26 °C (Additional files 6, 7: Table S4, S5). Common genes altered in all of these treatments were TcSYL_0078950 and TcSYL_0103620 annotated under the terms lipid metabolic process and oxidation-reduction process, respectively. In addition, genes involved in the synthesis of some amino acids, dependent on this metabolic pathway, such as arginine (TcSYL_0201940) for TcII at 27 °C and proline (TcSYL_0201810) for TcI at 28 °C, were downregulated (Additional file 6: Table S4). The downregulation of a gene associated with the tetrahydrofolate biosynthetic process (log2-fold_change: − 158.836, TcSYL_0078950) in TcI at 27 °C was also detected (Additional file 6: Table S4).

The coding transcript for the hexokinase enzyme (TcSYL_0169190-TcSYL_0169200) showed a decrease in expression for all of the treatments compared with the control. Similarly, the transcript for lactate/malate dehydrogenase with the alpha/beta C-terminal domain (TcSYL_0122290) was upregulated for all treatments except for TcII at 28 °C. Aldose 1-epimerase (TcSYL_0046290) was upregulated in MT exposed to 27 °C and in TcII at 28 °C. Similar results were found for the transcript encoding glucosamine-6-phosphate isomerases/6-phosphogluconolactonase, which was upregulated for TcI at 27 °C and TcII at 28 °C (TcSYL_0165520) (Additional files 6, 7: Table S4, S5). A more in-depth analysis of glucose metabolism revealed a decrease in expression levels for genes involved in the GO terms “glucose 6-phosphate metabolic process” and “cellular glucose homeostasis” for TcI at 28 °C and TcII at 27 °C; however, the genes for the same GO terms were upregulated for TcII at 28 °C (Additional file 6: Table S4). The transcript TcSYL_0000660 coding for 6-phosphofructo-2-kinase involved in the GO fructose metabolic process was upregulated in most of the treatments except for TcI at 27 °C (Additional files 6, 7: Table S4, S5).

### Oxidoreduction

One of the processes most affected by the exposure of MT to different temperatures was oxidoreduction. A total of 15 transcripts for TcI at 27 °C, 29 transcripts for TcI at 28 °C, 34 transcripts for TcII at 27 °C and 17 transcripts for TcII at 28 °C corresponded to downregulated genes involved in the reduction-oxidation process. In addition, a small number of upregulated genes related to the reduction-oxidation process was detected for TcI at 27 °C (5 transcripts), TcI at 28 °C (7 transcripts) and TcII at 27 °C (10 transcripts); however, 47 transcripts were detected for TcII at 28 °C (Additional files 6, 7, 8: Tables S4, S5, S6). The products for each of the downregulated and upregulated genes are available in Additional file 8: Table S6, including different types of oxidoreductases, peroxidases, hydrogenases and cytochrome B oxidases involved in this pathway.

TcI showed downregulation in the expression of genes mainly responsible for the management of oxidative stress in parasites incubated at 27 °C. Among these genes were quinone reductase (NADPH), fumarate reductase (NADH), and cytochrome *c* oxidase, as well as amino acid kinases and peptidases, which are indispensable for obtaining energy in the parasite during part of metacyclogenesis. TcI exposed to 28 °C showed a decrease in the expression of proteins responsible for the regulation of oxidative stress, similar to the results at 27 °C, but also showed decreased expression of other proteins such as cytochrome-b5 reductase and alcohol dehydrogenase (Additional file 8: Table S6), which indicates that the differential expression of these proteins may be a consequence of the exposure of MT to high temperatures.

### Proteins related to proteolysis

Proteins related to proteolysis play an important role in the regulation, maintenance, and progression of the life-cycle of *T. cruzi*. Our results revealed the regulation of genes coding for proteins of some important families related to these processes (Additional files 6, 7, 8: Tables S4, S5, S6). The predominant family of proteins that were regulated included leishmanolysin or GP63, and a total of 8 genes were downregulated and 4 were upregulated for TcI at 27 °C, 4 were downregulated and 3 were upregulated for TcI at 28 °C, no genes were downregulated but 4 were upregulated for TcII at 27 °C, and 3 were downregulated and 8 were upregulated for TcI at 28 °C (Additional files 6, 7: Table S4, S5). It is important to note that although genes encoding proteins within this family were both upregulated and downregulated, none of the genes ID showed both of these outcomes indicating the specific up- or downregulation of specific GP63 proteins (Additional file 9: Table S7).

The M32 metallopeptidase family of proteins showed different expression patterns between treatments. Two transcripts were upregulated for TcI at 27 °C and downregulated for TcI at 28 °C (TcSYL_0019490 and TcSYL_0138670), TcSYL_0138680 being the only gene that was downregulated for TcI at 28 °C. Regarding TcII, transcripts TcSYL_0019490 and TcSYL_0138670 were downregulated in MT exposed to both temperatures (Additional file 9: Table S7).

The calpain family of cysteine proteases was another family of proteins regulated in MT under different temperatures. The transcripts TcSYL_0080730, TcSYL_0080800, TcSYL_0080820 and TcSYL_0146610 were upregulated in TcI at 27 °C, whereas for TcII at 27 °C only the TcSYL_0146610 gene was upregulated, and TcII at 28 °C showed a decrease in the expression of TcSYL_0080820 and TcSYL_0146610 genes, and an increase in expression of TcSYL_0063550 (Additional file 9: Table S7).

Finally, both thermal treatments triggered the overexpression of ubiquitin-2-like Rad60 SUMO-like (TcSYL_0109300) protein in TcII. In addition, a series of proteases were expressed differentially in some of the treatments. When evaluating TcI at 28 °C, transcript TcSYL_0075250 coding for the cytosol aminopeptidase family was downregulated, whereas TcSYL_0202010 (M16C-associated peptidase) was upregulated (Additional file 6: Table S4). For TcII at 27 °C, TcSYL_0013180 (prolyl oligopeptidase family) was overexpressed. Finally, for TcII at 28 °C, TcSYL_0171200 (serine carboxypeptidase) was upregulated (Additional file 9: Table S7).

### Proteins related to phosphorylation

The transcripts TcSYL_0114970 (chromosome 18) and TcSYL_0171180 (chromosome 36), both annotated as protein kinase domains, were downregulated for TcI at 27 °C, and simultaneously, a total of 12 protein kinase domain transcripts were upregulated. For TcI at 28 °C, 6 genes coding for protein kinase domains were downregulated and 12 genes were upregulated. For TcII, the same genes were downregulated and 34 were upregulated in the MT exposed to 27 °C, and 16 were downregulated and 41 were upregulated in the MT exposed to 28 °C (Additional file 10: Table S8).

### Translation

One of the most disturbed processes when evaluating the GO for the DEGs in the MT exposed to different temperatures was translation. A total of 26 transcripts related to the process of translation were downregulated for TcI at 27 °C and 48 transcripts were downregulated for TcI at 28 °C, compared with 6 upregulated transcripts for 27 °C and 5 upregulated transcripts for 28 °C. The results obtained for TcII showed similar patterns, especially when evaluating MT exposed to 27 °C, when 24 genes were downregulated and 6 genes were upregulated. By contrast, for TcII at 28 °C, 8 genes were downregulated and 7 genes were upregulated (Additional file 6: Table S4, Additional file 7: Table S5).

The family of ribosomal proteins were the most affected transcripts in MT after exposure to high temperatures, with 26 transcripts upregulated for TcI at 27 °C, 44 transcripts upregulated for TcI at 28 °C, 18 transcripts upregulated for TcII at 27 °C and only two transcripts upregulated for TcII at 28 °C. The complete list of DEGs is available in Additional file 7: Table S5. Transcripts TcSYL_0010030 (KH domain) and TcSYL_0103320 (KOW motif) were downregulated in TcI at 28 °C and TcII at 27 °C, tRNA synthetases of class I (M) (TcSYL_0170460) was overexpressed in TcI for both treatments and two transcripts coding for tRNA synthetases of class I (I, L, M and V) (TcSYL_0090210 and TcSYL_0140390) and two transcripts coding for mitochondrial small ribosomal subunit Rsm22 (TcSYL_0048380 and TcSYL_0048390) were upregulated in TcI at 27 °C. For TcI at 28 °C, there was an increase in the expression of TcSYL_0166880 and TcSYL_0202360, both coding for tRNA synthetases of class II (D, K and N) (Additional file 11: Table S9). Finally, the upregulated genes in TcII at 27 °C encoded families of ribosomal proteins L4/L1 (TcSYL_0003330), S4 (TcSYL_0011150), L2 (TcSYL_0013860) and L14p/L23e (TcSYL_0046720), whereas these genes were downregulated in the other treatments. In addition, upregulation of the RNA polymerase I-associated factor PAF67 (TcSYL_0113260) was observed in TcII at 27 °C (Additional file 11: Table S9).

### Vesicle-mediated transport

Genes involved in vesicle-mediated transport were downregulated in three of the four evaluated treatments (TcI 27 °C, TcII 27 °C, TcII 28 °C) with respect to the control. The coding transcript for vesicle-mediated transport (TcSYL_0091630) was downregulated in TcII at 27 °C and 28 °C, as were genes involved in the transport of ions (TcSYL_0109340), metal ions (TcSYL_0178120), cations (TcSYL_0174330), chloride ions (TcSYL_0111950) and hydrogen ions (TcSYL_0181420). The transport of some macromolecules essential for the parasite was also affected in TcI at 27 °C and TcII at 28 °C, with a decrease in the expression of the genes TcSYL_0146190 (protein transport) and TcSYL_0047800 (nucleoside transmembrane transport) (Additional files 6, 7: Table S4, S5).

### A greater number of upregulated genes observed in TcII than in TcI when exposed to different temperatures

When DTUs were compared between the different temperature treatments, the ontology with the highest number of downregulated and upregulated genes was related to molecular processes. When comparing between DTUs at 26 °C, a total of 957 terms were downregulated in TcII compared to TcI and 810 terms were upregulated in the same comparison, this being the only temperature where the number of downregulated genes was higher than that of upregulated genes. A decrease in the expression of structural components of ribosomes and integral components of the membrane and cytoplasm was observed in TcII compared with TcI, in addition to an increase in the expression of genes that encode DNA binding proteins for this DTU (Additional file 8: Table S6). The results obtained at 27 °C revealed 1427 downregulated genes and 1274 regulated genes, and consistent with the results at 26 °C, there was a decrease in the expression of genes coding for integral components of the membrane and cytoplasm, in addition to an increase in the expression of genes encoding nucleic acid binding proteins (Additional file 12: Table S10). Finally, the highest temperature evaluated in this study (28 °C) showed a total of 1210 downregulated and 1943 upregulated terms between DTUs. Ontological terms related to proteolysis and integral membrane components were found downregulated for this analysis, whereas the upregulated ontological terms that contributed most to this comparison were related to DNA binding (Additional file 12: Table S10).

## Discussion

The results of this study show how slight changes in temperature affect the gene expression of two of the most important DTUs of *T. cruzi* (Fig. 2, Additional file 5: Table S3). Herein, we observed how temperature decreased the concentration of MT and slowed transformation, as evident when evaluating the EMD (Fig. 1). Similar results were observed when the effect of temperature on the metacyclogenesis of *T. cruzi* in *R. prolixus* was evaluated, confirming that the parasite maintains the same biological characteristics in studies both *in vivo* and *in vitro* [14]. When TcII was exposed to 28 °C, this temperature affected the concentration of MT when compared with 26 °C for the same DTU but it was significantly downregulated in TcI at 28 °C, potentially indicating resistance in the DTU of TcII at high temperatures. The EMD for TcII at 28 °C was lower than that of TcII at 26 °C, confirming the resistance of TcII when exposed to high temperatures (Fig. 1). Previous studies evaluating the metacyclogenesis process and presence of MTs *in vivo* showed similar results suggesting that TcII parasites have adapted to high temperatures [14]. Differences between these DTUs during metacyclogenesis have already been reported in other *in vitro* studies, where the concentration of MT was higher compared with TcI and TcIV DTUs following exposure to high temperatures [38]. Despite this, we observed regulation of the expression of different genes between parasites treated with different temperatures and between DTUs treated at the same temperature, with fewer genes regulated between DTUs treated at the same temperature, especially for 27 °C and 28 °C (Fig. 2 a, Additional file 5: Table S3). This characteristic indicates that at higher temperatures or even under stress conditions, the parasite could decrease the expression of single genes by DTUs and increase the expression of genes associated with cell stress for cell survival purposes; however, further in-depth studies are required to confirm this, that include a greater number of biological replicates.

The analyses performed here showed that chromosome 1 harbored the majority of DEGs (Fig. 2c). Interestingly, despite the fact that the length of this chromosome exceeds 3 Mb, it comprises a higher number of housekeeping genes, unlike other chromosomes that contain a large number of repetitive genes encoding surface proteins [39]. On the other hand, the presence in the *T. cruzi* genome of a compartment core that mainly includes conserved and disruptive genes that cover most of the repetitive coding sequences for surface proteins, influence the unequal grouping of these proteins throughout the genome and of course on each of the chromosomes [40]. Structural changes, including copy number variations (CNV) and single nucleotide polymorphisms, have been observed in response to environmental stimuli and as a consequence of genomic adaptations [41, 42]. Based on these results, we hypothesize that *T. cruzi* may display the same characteristic of genomic adaptation to the environment, and that consequently, in response to thermal stress, may generate structural variations as a result of genetic changes on chromosomes 1 and 3. The ability to generate mutations as a consequence of oxidative stress has already been proven in this parasite and oxidative stress is believed to be the main trigger for the generation of genetic mutations during metacyclogenesis of *T. cruzi*, indicating the ability of this parasite to adapt to environmental stresses [43]. However, genomic studies to assess the effect of temperature or other stress stimuli on the genome of this parasite are required to support this premise including a better annotation of the available *T. cruzi* genomes.

Genes involved in the glucose 6-phosphate metabolic process and cellular glucose homeostasis were also upregulated in TcI at 28 °C and TcII at 27 °C (Additional files 6, 7: Table S4, S5). One of the enzymes involved in this process, glucose-6-phosphate dehydrogenase, whose function is to catalyze one of the first reactions in the pentose phosphate pathway and consequently produce NADPH, plays an essential role during infection and the defense against oxidative stress, which is why it has also been used in the study of therapeutic targets [44, 45]. Therefore, the decrease in the expression of proteins involved in the processes in which tetrahydrofolate acid and glucose 6-phosphate play a role, may have a strong influence on the cell death that occurs in the MT of TcI at 28 °C and TcII at 27 °C, and also on the survival of TcII at 28 °C when these genes are upregulated.

Oxidative stress is associated with cell death; however, under normal conditions, *T. cruzi* has the ability to cope with oxidative stress by producing a large number of antioxidant proteins and DNA repair proteins [46–49]. Our results showed a decrease in the expression of a large number of genes involved in these processes, such as quinone reductase (NADPH), fumarate reductase (NADH), cytochrome *c* oxidase and alcohol dehydrogenase, with these genes being more highly expressed in parasites exposed to 27 °C (Additional file 8: Table S6). However, even more interesting is the massive increase in the number of downregulated genes relating to oxidative stress in TcI at 27 °C and 28 °C and in TcII at 27 °C, compared with the increase in upregulated genes related to this process in TcII at 28 °C (Additional file 8: Table S6). These findings may explain the decrease in the concentration of MT at these temperatures as a result of cell death due to uncontrolled oxidative stress and the possible resistance of MT of TcII at 28 °C when exposed to this same source of stress, potentially illustrating the ability of this parasite to manage oxidative stress and increase of expression of genes linked to this process.

The differential expression of protein kinases during metacyclogenesis has been reported [46]. In our study, an increase in the number of genes coding for these proteins was found at all of the temperatures tested for both DTUs when compared with the control; however, the most dramatic change was exhibited by TcII, with 34 and 41 upregulated genes being observed at 27 °C and 28 °C, respectively (Additional file 10: Table S8), indicating the strong influence of these proteins in this DTU as a response to thermal stress. However, the reference genome used here for mapping does not allow us to determine the type of kinases expressed; a more in-depth study of the sequences of differentially expressed transcripts may provide insight into the specific function of the proteins to be translated. The presence of mitogen-activated protein kinases (MAPKs) in *T. cruzi* and their participation in evasion of the immune system has been reported, and many of these proteins play a role the stress response in other eukaryotes. For example, the Smp38 MAPK has been reported to be involved in the regulation of homeostasis in *Schistosoma mansoni* under oxidative stress, which is one of the main stimuli of metacyclogenesis, and may not therefore be affected by exposure to high temperatures. Protein kinases with this type of function may be upregulated in TcII as a contingency mechanism for this type of cellular stress [50, 51].

One of the characteristics of metacyclogenesis is an increase in transcription and translation followed by a decrease in the forms of MT. This is a consequence of the exposure of *T. cruzi* to nutrient-deficient medium, which leads to higher energy requirements that are achieved mainly by protein degradation and post-translational regulation. We therefore propose that when parasites are subjected to similar types of stress, such as temperature, this same behavior may be triggered in the parasite [25, 52]. Our findings revealed a drastic decrease in the expression of a large number of genes coding for members of the ribosome family in three of the four thermal treatments (Additional file 11: Table S9). This indicated that the decrease in the expression of constitutive ribosomal proteins, and as a consequence the synthesis of new ribosomes, is related to the exacerbated decrease in translation in TcI at 27 °C and 28 °C and TcII at 27 °C and the presence of MT forms with these particular characteristics (Additional file 11: Table S9). The presence of ribosomal profiles in which different ribosomal proteins are combined, has already been observed in *Toxoplasma gondii* and *T. cruzi* as a mechanism for translational control between different stages and for the translation of virulence factors [53]. Studies where the expression of ribosomal proteins was analyzed showed the decrease in translation efficiency in MT compared to EP for the same strain; these results demonstrate the importance of these proteins in gene regulation between different stages of the parasite, and could give us an indication of the regulation of specific profiles of ribosomal proteins in response to any type of stress, especially considering the distinctive regulation of expression that trypanosomatids present compared to other eukaryotes [54]. The decrease in transcripts of ribosomal proteins found in our study may be related to a specific profile of translational regulation associated with the response to thermal stress; however, a more detailed study is needed to confirm this.

Metalloproteases (GP63) are a family of glycosylphosphatidylinositol (GPI)-labeled proteins present in trypanosomatids whose main function is associated with virulence. Transcriptomic analyzes have shown the presence of a group of these proteins that are expressed throughout the parasite life-cycle, while approximately the remaining 50% are stage-specific [28]. Suggesting specific functions between members of this protein family, the increase in the expression of GP63 in TcII could suggest the presence of a specific group of GP63, active in response to heat stress in this DTU, with a possible influence in quick response to stress conditions. On the other hand, the importance of GP63 has been observed during the adherence of *T. cruzi* to the intestine of the vector and the binding to host cells during infection in the host, considering our results, it could be inferred that TcII when exposed at high temperatures could acquire advantages in transmission and infection and, therefore, in the progression of its life-cycle [55–57]. However, a characterization study of the GP63 class expressed in TcII must be performed in order to know the type of response to thermal stress present in this DTU.

One of the limitations of this study was that the exposure of the parasite to different temperatures was only performed for one incubation time. This was because MT do not have the capacity for replication, so it was impossible to continue their culture in axenic medium. One of our results showed an increase in genes expressed from chromosome 1; however, complete genome analysis was not performed for the parasites exposed to different temperatures, which may have provided further insights. One of the characteristics of *T. cruzi* is that its genome is largely comprised of repetitive sequences that mostly code for families of multigenic proteins. This feature makes it difficult to assemble sequenced genomes and the only reference genome currently available is for the TcI DTU, which is at the level of chromosome assembly. Our study was conducted using this genome and this is why special care had to be taken when analyzing the differential expression of genes in TcII. In some cases, manual checks were needed to avoid bias, so the availability of a reference genome for this DTU would facilitate the analysis and assembly process for future analyses for TcII. Differences between strains of the same DTU have been observed at both genomic and biological levels; although our results cannot be fully extrapolated to all populations of TcI and TcII strains, they provide a first approach to a better comprehension of the influence of temperature stress on gene expression of MT that had not been previously evaluated. Future studies must be conducted to unravel the influence of temperature on gene expression including a greater number of strains by DTU.

## Conclusions

Our study showed that temperature affects *T. cruzi* I and II MT through regulation of the expression of genes coding for proteins involved in the metabolism of lipids and carbohydrates, the evasion of oxidative stress, proteolysis and phosphorylation processes, and also, the decrease of ribosomal proteins involved in translation, leading to cell death. However, DTU TcII exhibited greater resistance to thermal stress and greater survival, and an increase in genes linked to the handling of oxidative stress and the expression of the GP63 protein was detected. This could be due to an early response to these stress conditions in the TcII DTU; the results obtained for the count of MT where from day 6 at 27 °C and on day 5 at 28 °C showed a higher concentration of MT compared to TcI. The main objective of this study was to evaluate the changes in transcriptomic profiles in TcI and TcII MT when they are subjected to thermal stress. Subsequent studies should focus on assessing how transcriptomic profiles change by using specific temperatures to which the parasites are exposed during their passage through the vector. The presence of differential stress responses in these DTUs during emergence of MT could explain the biological differentiation exhibited by these DTUs in the host, and could also indicate a possible change in the distribution and epidemiology of these DTUs with environmental temperature increases.

## Supplementary information

**Additional file 1: Table S1.** The number of raw sequence reads for the fastaq files obtained from the sequencing of MTs.

**Additional file 2: Table S2.** Files of the *T. cruzi* reference genome Sylvio X10-2 with links for access to the fasta and gff *T. cruzi* Sylvio X10-2 reference files.

**Additional file 3: Figure S1.** RNA Quality analysis.

**Additional file 4: Figure S2.** Differential comparison between technical and biological replicates.

**Additional file 5: Table S3.** Differentially expressed genes for MT of TcI and TcII using the cuffdiff tool of cufflinks.

**Additional file 6: Table S4.** Gene ontology (GO) terms for the genes downregulated and upregulated for MT of TcI for both treatment temperatures.

**Additional file 7: Table S5.** Gene ontology (GO) terms for the genes downregulated and upregulated for MT of TcII for both treatment temperatures.

**Additional file 8: Table S6.** Oxidation reduction process. Gene IDs and the products for the downregulated and upregulated genes involved in oxidation reduction process for MT of TcI and TcII for both treatment temperatures.

**Additional file 9: Table S7.** Proteolysis process. Gene IDs and the products for the downregulated and upregulated genes involved in proteolysis process for MT of TcI and TcII.

**Additional file 10: Table S8.** Phosphorylation protein process. Gene IDs and the products for the downregulated and upregulated genes involved in phosphorylation protein process for MT of TcI and TcII.

**Additional file 11: Table S9.** Translation process. Gene IDs and the products for the downregulated and upregulated genes involved in translation process for MT of TcI and TcII.

**Additional file 12: Table S10.** Gene ontology (GO) terms for the downregulated and upregulated genes for the comparison between MT of TcI and TcII for each temperature.

## Data Availability

Data supporting the conclusions of this article are included within the article and its additional files. All data employed in this manuscript are available in the European Nucleotide Archive (ENA) under PRJEB33521 project (https://www.ebi.ac.uk/ena/data/view/PRJEB33521).

## Abbreviations

DTU: discrete typing unit
MT: metacyclic trypomastigote
EP: epimastigotes
cAMP: cyclic amino acid phosphate
RNA: ribonucleic acid
mRNA: messenger ribonucleic acid
GP: glicoprotein
BMD: the beginning of metacyclogenesis day
DPC: days post-culture
GO: gene ontology
