# Supplementary material for: Slight temperature changes cause rapid transcriptomic responses in Trypanosoma cruzi metacyclic trypomastigotes
Source: Parasit Vectors. 2020 May 14;13:255. doi: 10.1186/s13071-020-04125-y (PMC7226949; doi:10.1186/s13071-020-04125-y)
Supplement: Supplementary file 2 — Additional file 2: Table S2. Files of the T. cruzi reference genome Sylvio X10-2 with links for access to the fasta and gff T. cruzi Sylvio X10-2 reference files. [file 13071_2020_4125_MOESM2_ESM.docx]

**Additional Table S2: Links *T. cruzi* Sylvio X10-2 reference files**

| **File** | **Link** |
| --- | --- |
| **Genome** | <https://tritrypdb.org/common/downloads/Current_Release/TcruziSylvioX10-1/fasta/data/> |
| **Annotated file** | <https://tritrypdb.org/common/downloads/Current_Release/TcruziSylvioX10-1/gff/data/> |
