# Supplementary figures and images for: Slight temperature changes cause rapid transcriptomic responses in Trypanosoma cruzi metacyclic trypomastigotes
Source: Parasit Vectors. 2020 May 14;13:255. doi: 10.1186/s13071-020-04125-y (PMC7226949; doi:10.1186/s13071-020-04125-y)

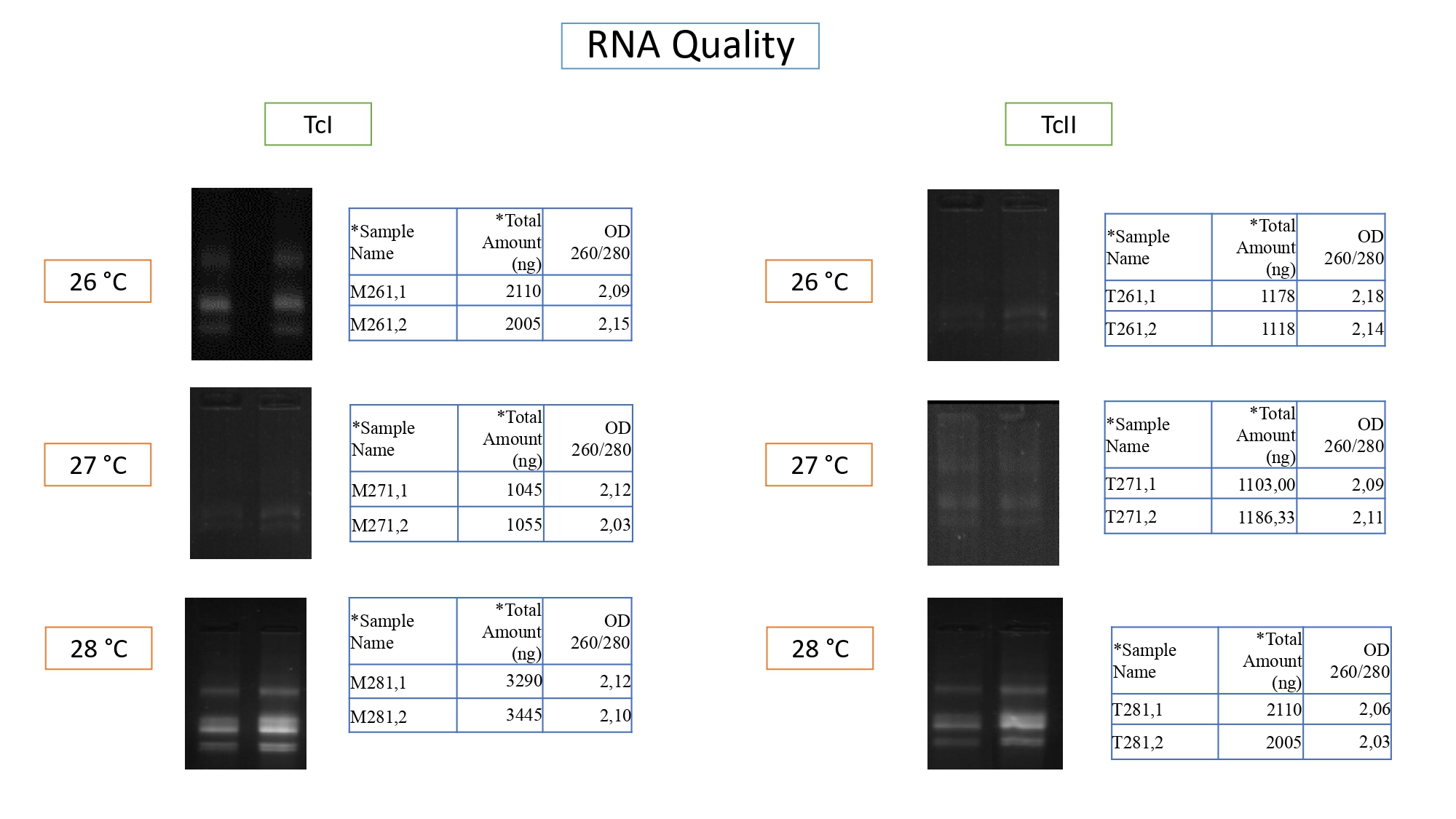

Supplement: Supplementary file 3 — Additional file 3: Figure S1. RNA Quality analysis. [file 13071_2020_4125_MOESM3_ESM.jpg]

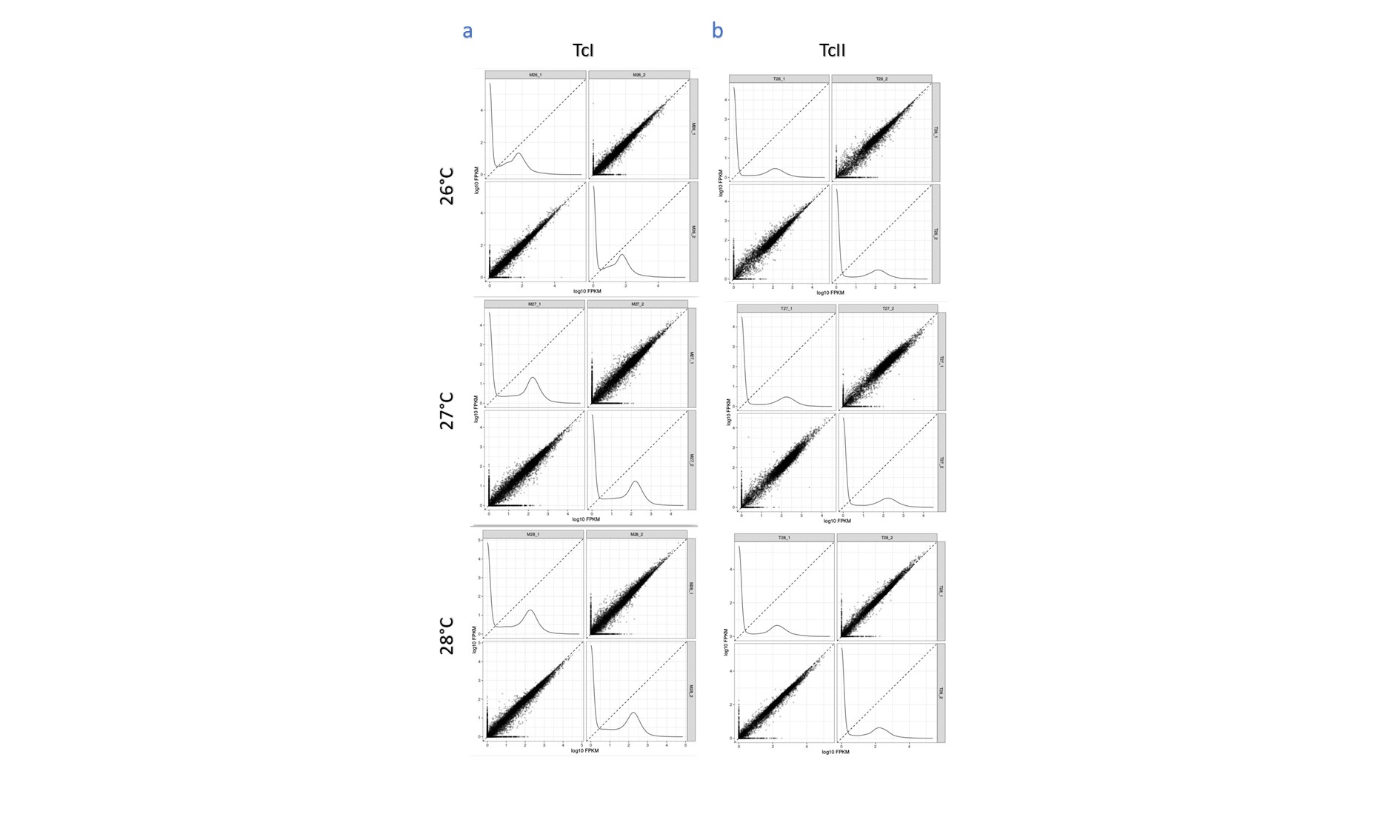

Supplement: Supplementary file 4 — Additional file 4: Figure S2. Differential comparison between technical and biological replicates. [file 13071_2020_4125_MOESM4_ESM.jpg]
